# Immunological Factors in Recurrent Pregnancy Loss: Mechanisms, Controversies, and Emerging Therapies

**DOI:** 10.3390/biology14070877

**Published:** 2025-07-17

**Authors:** Efthalia Moustakli, Anastasios Potiris, Athanasios Zikopoulos, Eirini Drakaki, Ioannis Arkoulis, Charikleia Skentou, Ioannis Tsakiridis, Themistoklis Dagklis, Peter Drakakis, Sofoklis Stavros

**Affiliations:** 1 Laboratory of Medical Genetics, Faculty of Medicine, School of Health Sciences, University of Ioannina, 451 10 Ioannina, Greece; thaleia.moustakli@gmail.com; 2 Third Department of Obstetrics and Gynecology, University General Hospital “ATTIKON”, Medical School, National and Kapodistrian University of Athens, 124 62 Athens, Greece; thanzik92@gmail.com (A.Z.); garkoylis@hotmail.com (I.A.); pdrakakis@med.uoa.gr (P.D.); sfstavrou@med.uoa.gr (S.S.); 3 First Department of Obstetrics and Gynecology, Alexandra Hospital, Medical School, National and Kapodistrian University of Athens, 115 28 Athens, Greece; eirinidrak@med.uoa.gr; 4 Department of Obstetrics and Gynecology, Medical School, University of Ioannina, 451 10 Ioannina, Greece; haraskentou@uoi.gr; 5 Third Department of Obstetrics and Gynecology, General Hospital Ippokratio, Medical School, Aristotle University of Thessaloniki, 546 42 Thessaloniki, Greece; iotsakir@gmail.com (I.T.); tdagklis@gmail.com (T.D.)

**Keywords:** immunological factors, recurrent pregnancy loss (RPL), assisted reproductive technologies (ART), cytokines, emerging therapies

## Abstract

Immunological factors have gained growing recognition as key contributors to recurrent pregnancy loss (RPL) after in vitro fertilization (IVF), representing a major challenge in reproductive medicine. RPL affects approximately 1–2% of women trying to conceive naturally and up to 10–15% of those undergoing IVF, where overall success rates remain around 30–40% per cycle. An imbalance in maternal immunological tolerance toward the semi-allogeneic fetus during pregnancy may lead to miscarriage and implantation failure. IVF-related ovarian stimulation and embryo modification offer additional immunological complications that can exacerbate existing immune dysregulation. Recent advances in reproductive immunology have significantly deepened our understanding of the immune mechanisms underlying RPL following IVF, particularly highlighting the roles of regulatory T cells (T regs), natural killer cells, cytokine dysregulation, and disruptions in maternal–fetal immune tolerance. In order to better customize therapies, this evaluation incorporates recently discovered immunological biomarkers and groups patients according to unique immune profiles. Beyond conventional treatments like intralipid therapy and intravenous immunoglobulin, it also examines new immunomodulatory medications that target certain immune pathways, such as precision immunotherapies and novel cytokine modulators. We also discuss the debates over immunological diagnostics and therapies, such as intralipid therapy, intravenous immunoglobulin, corticosteroids, and anticoagulants. The heterogeneity of patient immune profiles combined with a lack of strong evidence highlights the imperative for precision medicine to improve therapeutic consistency. Novel indicators for tailored immunotherapy and emerging treatments that target particular immune pathways have encouraging opportunities to increase pregnancy success rates. Improving management approaches requires that future research prioritize large-scale clinical trials and the development of standardized immunological assessments. This review addresses the immunological factors in RPL during IVF, emphasizing underlying mechanisms, ongoing controversies, and novel therapeutic approaches to inform researchers and clinicians.

## Simple Summary

Immunological factors are key contributors to recurrent pregnancy loss (RPL) and a major challenge in reproductive medicine. RPL affects about 1–2% of women trying to conceive naturally and up to 10–15% of women undergoing in vitro fertilization (IVF). Despite advances, IVF success rates remain around 30–40% per cycle, with immune-related causes being a significant factor in failed pregnancies. Recent advances in reproductive immunology have significantly deepened our understanding of the immune mechanisms underlying RPL following IVF, particularly highlighting the roles of regulatory T cells (T regs), natural killer cells, cytokine dysregulation, and disruptions in maternal–fetal immune tolerance. Important debates about diagnostic standards and the effectiveness of immunomodulatory therapies continue despite progress in identifying important immunological players. There are encouraging opportunities to improve outcomes for impacted couples with emerging medicines that target particular immunological pathways and use individualized techniques.

## 1. Introduction

RPL is typically defined as the loss of two or more pregnancies before 20 weeks of gestation and affects approximately 1–2% of women attempting to conceive [1]. It is an extremely disturbing reproductive disorder with significant psychological, social, and financial burdens on the families involved. The recurrent clinical conundrum in reproductive medicine arises from the inability of standard diagnostic techniques to identify an underlying etiology in up to 50% of cases of recurrent pregnancy loss, with recent studies reporting that a significant proportion of these losses involve euploid conceptions [2,3]. Diagnostic imprecision complicates the creation of therapy regimens of efficacy and the provision of thorough patient counseling [4].

The development and refining of assisted reproductive technologies (ART), particularly IVF, has transformed infertility care, allowing many couples to overcome previously insurmountable barriers to conception [5]. Nonetheless, IVF success rates remain suboptimal, with recurrent implantation failure and early pregnancy loss continuing to pose major challenges. Although immunological factors have long been hypothesized to contribute to these failures, recent evidence has questioned their clinical applicability, particularly in patients with unexplained RPL [6,7].

**Figure 1:**
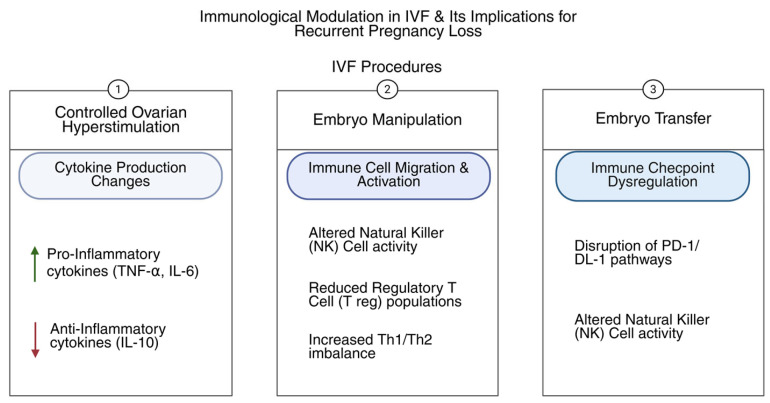
Schematic representation of immunological changes associated with IVF procedures.

Pregnancy represents an immunological challenge, requiring the maternal immune system to tolerate the semi-allogeneic fetus carrying paternal antigens while maintaining effective defense against pathogens. Intricate, strictly controlled interactions between cytokines, the maternal decidua, and innate and adaptive immune cells preserve this balance [8]. Aberrant immune activation arising from disruptions in immunoregulatory mechanisms can lead to placental dysfunction, implantation failure, and pregnancy loss [9] (Figure 1).

Further degrees of intricacy are added to this immune milieu by IVF. Controlled ovarian hyperstimulation, along with embryo manipulation and transfer procedures, can modulate the endometrial immune milieu by impacting cytokine production, immune cell migration, and the regulation of immune checkpoint molecule expression [10,11,12]. Infertility and repeated miscarriages among IVF patients are frequently linked to underlying immune diseases, making targeted immunological diagnoses and treatments crucial [13].

The exact mechanisms by which immunological dysregulation leads to RPL in IVF are not well understood, although interest in the topic has increased [14]. Despite the use of diverse diagnostic approaches to detect immunological disorders, consensus on standardized testing protocols and result interpretation criteria remains a subject of ongoing debate [15]. There is continuous discussion on the safety and effectiveness of different therapeutic approaches, ranging from sophisticated immunomodulatory therapies to widespread immunosuppression [16].

Although RPL happens with both natural conception and IVF, the complexity of the immune components involved varies [17]. Hormonal imbalances, genetic abnormalities, or immunological dysregulation of tolerance to the fetus are the main causes of natural conception-related RPL. By altering the uterine immunological environment through ovarian stimulation, embryo culture, and transfer procedures, IVF, however, presents new immune problems. These variables can change checkpoint regulation, cytokine levels, and immune cell behavior, which can impair immunological tolerance and raise the risk of miscarriage in IVF patients [18]. In IVF-related RPL, this complexity highlights the need for more accurate immunological diagnosis and individualized treatment [13].

The immunological reasons behind RPL in IVF, developing and innovative immunotherapeutic treatments, and current diagnostic issues and debates will all be methodically covered in this study [19]. To provide a comprehensive overview that informs future research and clinical practice in this evolving field, this review systematically addresses these critical aspects.

The objective of this manuscript is to examine ongoing debates in immune assessment and management, systematically review the immunological factors contributing to recurrent pregnancy loss after IVF, and evaluate novel treatment options that could improve reproductive success. This review aims to inform research strategies and deepen understanding in this developing field by combining molecular insights with clinical evidence.

## 2. Background: Immune Mechanisms in Pregnancy and RPL

RPL is a multifactorial syndrome resulting from the complex interplay among maternal, embryonic, and environmental factors [19,20]. Additional immunological hurdles are introduced by IVF in comparison to normal conception. Increased hormone levels brought on by controlled ovarian stimulation can have an impact on immune cell populations such as uterine natural killer cells and Tregs [21]. By avoiding maternal signals and normal embryonic selection, embryo cultivation and transfer may expose more people to embryos with lower immunological compatibility [22]. Furthermore, local inflammation and changes in endometrial cytokine profiles may result from the mechanical procedure of embryo transfer. These factors collectively create a distinct immunological environment in IVF pregnancies [23]. The distinct physiological environment in IVF procedures, as opposed to natural conception, introduces additional complexities that may alter the immunological processes critical for achieving and maintaining a successful pregnancy [24]. Hormonal changes brought on by the ovarian stimulation procedures used in IVF can affect the numbers and functions of immune cells, including Tregs and uterine natural killer (uNK) cells [25]. Moreover, embryo culture and transfer bypass natural selection mechanisms, potentially increasing the likelihood of uterine exposure to embryos with suboptimal immunological compatibility [26].

Pregnancy induces complex immunological adaptations in the mother, involving a diverse array of immune cells and cytokines that promote placental development and foster fetal tolerance [8]. Tregs, dendritic cells, macrophages, uNK cells, and cytokines that counterbalance pro- and anti-inflammatory cues are important participants. Recurrent pregnancy loss has been linked to dysregulation of these factors, especially during IVF, when immunological problems may be exacerbated [27,28]. The main immune cells and cytokines involved in a typical pregnancy are enumerated in Table 1, along with information on how their malfunction affects IVF pregnancy loss.

Disruptions in any of these elements can compromise the precise immunological balance critical for implantation and proper placental formation [35]. For instance, RPL has been linked to decreased Treg numbers or function, increased NK cell cytotoxic activity, and a skew of T helper cell subsets toward pro-inflammatory characteristics [36]. In IVF patients, immunological disturbances may be more pronounced or variable due to patient heterogeneity, repeated embryo manipulations, and the use of hormone therapies [6].

Assessing immunological factors in cases of RPL remains a significant clinical challenge. NK cell assays and cytokine profiling are examples of diagnostic techniques that are not standardized, and immune characteristics vary with the menstrual cycle and pregnancy status [37]. Further supporting the notion that RPL constitutes a syndrome with multiple immunopathogenic pathways, rather than a single disease entity, is the wide spectrum of immunological abnormalities observed among affected individuals [38]. Developing more accurate diagnostic methods and focused treatments requires an understanding of these immunological foundations, which will ultimately improve the results for women who experience RPL during IVF cycles [39].

## 3. Immunological Mechanisms in RPL During IVF

Successful pregnancies depend on a well-managed uterine immunological milieu, which is necessary for the mother’s immune system to maintain tolerance toward the semi-allogeneic fetus. Disturbances in these immune systems can compromise implantation and fetal development in RPL, especially during IVF cycles. Understanding the intricate immunological interactions involved is essential for identifying potential causes of pregnancy failure [40].

### 3.1. Maternal–Fetal Immune Tolerance

Throughout pregnancy, the maternal immune system must sustain tolerance to fetal antigens, avoiding rejection and thereby establishing a distinct immunological condition [41]. Tregs are pivotal in this process through the secretion of immunosuppressive cytokines, including transforming growth factor-beta (TGF-β) and interleukin-10 (IL-10), which promote the establishment of immunological tolerance. By inhibiting cytotoxic responses and suppressing effector T cell growth, fetal rejection is avoided, and the vascular remodeling required for the placenta is supported. Studies have shown that women with RPL exhibit reduced Treg counts and impaired Treg function, potentially leading to diminished immune tolerance and a heightened risk of miscarriage [14].

### 3.2. Innate Immunity and NK Cells

During early pregnancy, uNK cells are the main immune cells in the decidua and are distinct from peripheral NK cells in both phenotype and function. Through the secretion of angiogenic factors and the regulation of trophoblast invasion, uNK cells contribute to placental development [42]. They facilitate spiral artery remodeling and modulate trophoblast migration, processes that, if dysregulated, can result in shallow implantation and poor placentation associated with miscarriage. However, abnormal uNK cell activation or heightened cytotoxicity has been linked to RPL and implantation failure. In RPL, elevated peripheral NK cell activity has been proposed as a marker of immunological dysfunction, although this remains debatable due to variability in assessment techniques [43].

### 3.3. Adaptive Immunity and Autoimmunity

RPL is significantly influenced by autoimmune variables, particularly when antiphospholipid antibodies (aPL) and other autoantibodies are present. These autoantibodies can disrupt placental function and encourage thrombosis [44]. Moreover, patients with RPL exhibit an imbalance in T helper (Th) cell subsets, characterized by a shift toward a pro-inflammatory Th1 and Th17 profile. This cytokine environment fosters inflammation and cytotoxic responses that negatively impact embryo implantation and its subsequent maintenance [45].

### 3.4. Inflammatory Cytokines and Chemokines

Cytokines serve as key modulators of the immune response throughout pregnancy. Negative pregnancy outcomes have been linked to elevated levels of pro-inflammatory cytokines, including interleukin-6 (IL-6), interferon-gamma (IFN-γ), and tumor necrosis factor-alpha (TNF-α). By disrupting the balance between Th1 and Th2 immune responses, essential for sustaining early pregnancy, these cytokines enhance local inflammation and hinder trophoblast invasion. These cytokines can cause local inflammation, interfere with trophoblast invasion, and reduce endometrial receptivity, all of which can lead to IVF cycle pregnancy loss [46].

### 3.5. Genetic and Epigenetic Influences on Immune Regulation

According to this research, some women may be more susceptible to immunologically mediated RPL due to genetic variations that impact immune regulatory genes [47]. Gene expression and immunological responses required for pregnancy maintenance are largely regulated by epigenetic modifications, such as DNA methylation changes in immune cells. These factors could underlie individual variability in both the effectiveness of immunotherapies and the establishment of immunological tolerance [48].

Throughout implantation, placentation, and the first few months of pregnancy, immune patterns change and are dynamic. During implantation, trophoblast invasion and tissue remodeling are first facilitated by a pro-inflammatory milieu. To promote fetal growth and avoid immunological rejection by the mother, this is followed by a shift to an anti-inflammatory, tolerogenic state during placentation [8]. Pregnancy loss may be caused by dysregulation in the onset or severity of these immunological changes. Understanding the immunological origins of RPL and maximizing the timing of immunotherapies, particularly in IVF settings, depends on the recognition of these temporal immune alterations [49].

### 3.6. Uterine Microbiome and Endometrial Receptivity

Recent research indicates that, especially when it comes to RPL and IVF, the uterine microbiota may have an impact on reproductive outcomes. Impaired implantation and a higher risk of miscarriage have been linked to endometrial flora dysbiosis, which is typified by an imbalance in Lactobacillus-dominated communities [50]. Through changes in cytokine profiles and compromised endometrial receptivity, this dysbiotic condition can impair local immunological responses [51]. Recent research suggests that individuals with a dysbiotic endometrial microbiome may become pregnant more frequently than those with a normal microbiome following targeted antibiotic and probiotic treatments [52]. Furthermore, a promising tactic to maximize implantation success in IVF cycles is individualized endometrial receptivity testing that combines immunological profile and microbiome investigation.

## 4. Controversies in Immunological Assessment and Treatment

Significant debates still surround diagnostic assessment and treatment approaches, despite progress in our understanding of the immunological mechanisms causing RPL during IVF. These disagreements originate from the limited evidence base for different immunotherapies, variability in study outcomes, and inconsistencies in immunological testing methodologies [53,54].

### 4.1. Diagnostic Challenges

One of the primary challenges in the management of immune-related RPL is a lack of standardized, globally recognized diagnostic criteria. As an example, NK cell activity measurement remains extremely controversial [55]. It is unclear which of the cut-off values distinguish abnormally elevated or depressed levels, and cytotoxicity assays and NK counts in peripheral blood cannot for certain be translated as a measurement of uterine NK functional activity [56]. Clinical decision-making is further hampered by variability in sensitivity and specificity of autoantibody assays, including antiphospholipid antibodies [57]. Cytokine profiling and immune phenotyping, as promising as they are, are primarily research tools without clinical guidelines yet in existence. The multiplicity of patient groups, as well as immune response variability during the course of a menstrual cycle, creates complexity in interpreting results [58].

### 4.2. Debate over the Role of Immune Dysregulation

The exact contribution of immune dysregulation in RPL is disputed. While some studies support findings of elevated NK cell activity and pro-inflammatory cytokines, others have not validated those findings or proven causation [59]. Other researchers have held that observed immune abnormalities are epiphenomena, not immediate predictors of loss of miscarriage [60]. Moreover, protective and harmful effects of immune activation act within an environment where immune systems both resist infectious agents and maintain tolerance. Such an environment makes therapeutic targeting of the immune system without compromise of host defense difficult [61,62].

### 4.3. Therapeutic Controversies

Immune-related RPL remains controversial due to a paucity of high-quality evidence and mixed clinical effects. Immunomodulatory agents, among which corticosteroids, IVIG, intralipid infusions, low-dose aspirin, and heparin have been utilized, possess mixed risk and efficacy profiles [49] (Table 2). While corticosteroids, by virtue of their immunosuppressive effects, have disadvantages in terms of side effects and efficacy, IVIG has proven efficacy in selected studies but is tainted by high costs and a lack of large, blinded, randomized trials [63,64]. Intralipid infusion, directed against NK-cell activity, remains experimental with inconclusive data [65]. Aspirin and heparin, anticoagulants with mainly possible immune-modulation effects, are usually reserved for antiphospholipid syndrome or thrombophilia. As a result of risks for overtreatment and side effects, careful patient selection is necessary, and additional studies are required for subgroups who would optimally benefit from immunotherapy [66,67].

## 5. Emerging Therapies and Future Directions

As knowledge of the immunological basis for RPL in IVF increases, new therapeutic approaches are in progress aimed at enhancing pregnancy outcomes. The newer therapies, as they continue to evolve, promise a more precise attack on unique immune mechanisms, beyond generalized immunosuppression, in favor of personalized immunomodulation [6,74].

### 5.1. Novel Immunomodulatory Agents

Molecules targeting certain inflammatory cytokines and immune checkpoints have shown promise in preclinical studies and phase I clinical trials [75]. In RPL cases with significant pro-inflammatory cytokine production, for example, anti-TNF-α medications, which are commonly used in autoimmune disorders, may reduce harmful inflammation, especially when TNF-α is linked to poor endometrial receptivity or embryo rejection [76,77]. Furthermore, it has been suggested that drugs that target IL-17 or other cytokines in the Th17 pathway can help women with RPL regain the Th17/Treg balance, which is commonly upset [78].

The potential of emerging possibilities like IL-1β antagonists and tocilizumab (anti-IL-6R) to lessen immune-mediated implantation failure is also being studied. Nonetheless, a crucial topic of further investigation is the safety of cytokine-targeting treatments in the early stages of pregnancy [79,80].

### 5.2. Cell-Based Therapies

A state-of-the-art treatment for RPL in women is adaptive transfer of Tregs, which restores immunological tolerance [81]. Preclinical models show that Treg infusion can promote implantation and fetal survivability by inhibiting dysregulated immune activation [82]. The use of low-dose IL-2 for in vivo Treg multiplication and improvements in GMP-compliant protocols are being intensively investigated to overcome logistical obstacles, but clinical applicability is still restricted by difficulties with Treg isolation, ex vivo expansion, and delivery [83].

Mesenchymal stromal cells (MSCs), which have immunomodulatory effects and may increase endometrial receptivity, are also being studied for their potential as a treatment [84].

### 5.3. Personalized Immunotherapy

With RPL immune dysregulation heterogeneity, there is a call for strategies of precision medicine [13]. Complex immune profiling technologies, including high-dimensional flow cytometry, single-cell transcriptomics, and proteomics, are increasingly capable of defining patient-specific immune signatures [85].

These technologies are being integrated with machine-learning programs to group immune phenotypes and predict therapeutic responses, such that individualized immunotherapy regimens with optimal efficacy and minimum systemic immunosuppression are obtainable [86]. Such an individualized strategy would most probably discriminate those women who would be likely to benefit from immune-targeted therapies and those who would not be likely to by such treatment regimens [87].

### 5.4. Immunogenetics and Biomarker Discovery

Genomic technologies are elucidating mutations and polymorphisms of immune-regulatory genes, e.g., FOXP3, HLA-G, and CTLA-4, that might predispose to RPL [88]. There is also promise for non-invasive biomarkers for predicting immune dysfunction and monitoring response to treatment by DNA methylation patterns and circulating microRNAs.

Efforts are being made now to validate these markers in large cohorts and to introduce them in clinical practice for early prediction of risk and optimization of treatment [89,90].

### 5.5. Non-Invasive Immune Monitoring

Liquid biopsy techniques that analyze circulating immune cells, cytokines, and extracellular vesicles have the potential for real-time, non-invasive monitoring of the maternal immune environment [91]. Liquid biopsy technologies allow for real-time, non-invasive assessment of the maternal immune milieu [92]. Via circulating immune cells, soluble cytokines, and exosomes, clinicians can observe immune status dynamically before and during IVF cycles. This could be used to guide the timing and vigor of immunotherapy to enhance outcomes and reduce overtreatment.

With these tools, immune adaptations during implantation are also made apparent, and novel insight is achieved for early pregnancy failure mechanisms [93,94].

### 5.6. Ongoing Clinical Trials and Research Gaps

There are several clinical trials in progress where novel immunotherapy and diagnostic modalities are being investigated in IVF women with RPL [95]. Examples include trials of immune checkpoint inhibitors, transfer of intrauterine immunoglobulins, and cytokine profiling of endometrium within IVF programs (e.g., NCT04643117, NCT05422170). These are interesting projects, but most are in early stages with limited sample numbers and inconsistently defined inclusion criteria [96]. For example, compared to a placebo, an interim analysis of study NCT04643117, which assessed intrauterine immunoglobulin transfer, indicated higher implantation rates; large, more rigorously powered investigations are needed to confirm these results.

**Figure 2:**
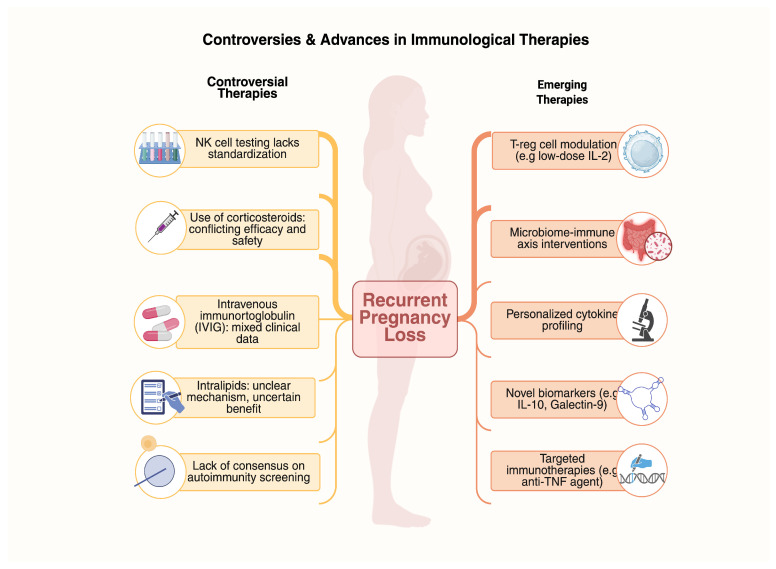
Comparative schematic of controversial versus emerging immunological therapies for RPL during IVF. The left panel lists therapies with limited or conflicting evidence, while the right panel shows newer approaches under investigation.

For the establishment of robust, evidence-based recommendations, multicenter randomized controlled trials of large size are required. More studies in the future would be ideal to clarify the complex interplay of immune, genetic, hormonal, and environmental factors in RPL pathophysiology, ideally by collaborative interactions across disciplines of immunology, reproductive medicine, and computational biology [97]. Figure 2 presents schematically the controversial versus emerging immunological therapies for RPL during IVF.

## 6. Conclusions

IVF-related RPL is still a complex clinical problem, with immunological variables being a crucial but poorly understood contributing element. Pregnancy failure can result from disturbances in innate and adaptive immune processes, and effective implantation and pregnancy maintenance depend on the delicate balance of maternal–fetal immunological tolerance [98]. Important debates about diagnostic standards and the effectiveness of immunomodulatory therapies continue despite progress in identifying important immunological players, such as cytokine networks, natural killer cells, and Tregs [99,100].

There are encouraging opportunities to improve outcomes for impacted couples with emerging medicines that target particular immunological pathways and use individualized techniques. To verify these interventions and create uniform protocols, however, strong clinical data are required. Multicenter RCTs should be given top priority in future research in order to standardize functional assays for NK cells, which currently exhibit significant variation throughout laboratories, and assess cytokine profiling as a diagnostic tool. Furthermore, to provide uniformity in clinical practice, immunological diagnostic criteria and treatment modalities must be harmonized.

To overcome these obstacles and enable integrative, precision-based care, cooperation between immunology, reproductive medicine, obstetrics, and bioinformatics will be necessary. In this developing sector, such multidisciplinary efforts are a vital call to action for improving patient outcomes and scientific understanding.

## Figures and Tables

**Table 1:** This table summarizes the roles of key immune components in maintaining immune tolerance and supporting placental development during normal pregnancy. It also outlines how dysregulation of these components is associated with RPL and impaired outcomes in IVF.

Immune Component	Role in Normal Pregnancy	Dysregulation Associated with RPL and IVF
Uterine Natural Killer (uNK) Cells [29]	Promote trophoblast invasion and vascular remodeling; maintain immune tolerance at the maternal–fetal interface	Increased cytotoxicity or abnormal activation linked to implantation failure and miscarriage
Regulatory T Cells (Tregs) [30]	Suppress maternal immune response to fetal antigens; maintain tolerance	Reduced number/function leads to loss of immune tolerance, increased inflammation
Macrophages [31]	Tissue remodeling, phagocytosis of apoptotic cells, immune regulation	Altered polarization (M1/M2 Macrophages imbalance) contributes to pro-inflammatory environment
Dendritic Cells [32]	Antigen presentation and immune modulation	Dysregulated antigen presentation can provoke immune rejection of the fetus
Cytokines (e.g., IL-10, TGF-β) [33]	Anti-inflammatory cytokines support tolerance and placental development	Decreased levels shift balance toward pro-inflammatory cytokines (e.g., TNF-α, IL-17) implicated in pregnancy loss
Cytokines (e.g., TNF-α, IL-17) [34]	Typically regulated to prevent excessive inflammation	Elevated levels promote cytotoxicity and tissue damage leading to miscarriage

**Table 2:** Summary of commonly used immunomodulatory therapies for immune-related recurrent pregnancy loss (RPL), their proposed mechanisms, evidence status, and limitations.

Therapy	Proposed Mechanism	Evidence Summary	Limitations/Challenges
Corticosteroids [68,69]	Immunosuppression	Some benefit in small studies	Side effects; inconsistent efficacy
IVIG [70]	Immunomodulation	Positive results in some trials	High cost; lack of large RCTs
Intralipid [71]	Suppression of NK cell activity	Experimental; mixed clinical results	Limited evidence; experimental status
Aspirin [72]	Anticoagulant, immune modulation	Useful in thrombophilia and APS	Limited to specific patient groups
Heparin [73]	Anticoagulant, immune modulation	Beneficial in APS and thrombophilia	Restricted to diagnosed cases; bleeding risk

IVIG: Intravenous immunoglobulin, RCT: Randomized Controlled Trial, APS: Antiphospholipid Syndrome.

## Data Availability

No new data were created or analyzed in this study. Data sharing is not applicable to this article.

## References

[1] Bender Atik R., Christiansen O.B., Elson J., Kolte A.M., Lewis S., Middeldorp S., McHeik S., Peramo B., Quenby S., Eshre Guideline Group on RPL (2023). ESHRE guideline: Recurrent pregnancy loss: An update in 2022. Hum. Reprod. Open.

[2] Sadeghi M.R. (2016). ART Strategy for Treatment of Recurrent Pregnancy Loss: Isn’t It Better to Forget?. J. Reprod. Infertil..

[3] Arnadottir G.A., Jonsson H., Hartwig T.S., Gruhn J.R., Moller P.L., Gylfason A., Westergaard D., Chan A.C., Oddsson A., Stefansdottir L. (2025). Sequence diversity lost in early pregnancy. Nature.

[4] Roosan D., Padua P., Khan R., Khan H., Verzosa C., Wu Y. (2024). Effectiveness of ChatGPT in clinical pharmacy and the role of artificial intelligence in medication therapy management. J. Am. Pharm. Assoc..

[5] Mackay A., Taylor S., Glass B. (2023). Inequity of Access: Scoping the Barriers to Assisted Reproductive Technologies. Pharmacy.

[6] Garmendia J.V., De Sanctis C.V., Hajduch M., De Sanctis J.B. (2025). Exploring the Immunological Aspects and Treatments of Recurrent Pregnancy Loss and Recurrent Implantation Failure. Int. J. Mol. Sci..

[7] Potiris A., Perros P., Drakaki E., Mavrogianni D., Machairiotis N., Sfakianakis A., Karampitsakos T., Vrachnis D., Antonakopoulos N., Panagopoulos P. (2024). Investigating the Association of Assisted Reproduction Techniques and Adverse Perinatal Outcomes. J. Clin. Med..

[8] Balasundaram P., Farhana A. (2025). Immunology at the Maternal-Fetal Interface.

[9] Geldenhuys J., Rossouw T.M., Lombaard H.A., Ehlers M.M., Kock M.M. (2018). Disruption in the Regulation of Immune Responses in the Placental Subtype of Preeclampsia. Front. Immunol..

[10] Vani V., Vasan S.S., Adiga S.K., Varsha S.R., Seshagiri P.B. (2023). Molecular regulators of human blastocyst development and hatching: Their significance in implantation and pregnancy outcome. Am. J. Reprod. Immunol..

[11] Cherouveim P., Mavrogianni D., Drakaki E., Potiris A., Zikopoulos A., Papamentzelopoulou M., Kouvoutsaki K., Machairiotis N., Karampitsakos T., Skentou C. (2023). ANRIL rs4977574 Gene Polymorphism in Women with Recurrent Pregnancy Loss. J. Clin. Med..

[12] Patronia M.M., Potiris A., Mavrogianni D., Drakaki E., Karampitsakos T., Machairoudias P., Topis S., Zikopoulos A., Vrachnis D., Moustakli E. (2024). The Expression of microRNAs and Their Involvement in Recurrent Pregnancy Loss. J. Clin. Med..

[13] Motlagh Asghari K., Novinbahador T., Mehdizadeh A., Zolfaghari M., Yousefi M. (2024). Revolutionized attitude toward recurrent pregnancy loss and recurrent implantation failure based on precision regenerative medicine. Heliyon.

[14] Uta C., Tirziu A., Zimbru E.L., Zimbru R.I., Georgescu M., Haidar L., Panaitescu C. (2024). Alloimmune Causes of Recurrent Pregnancy Loss: Cellular Mechanisms and Overview of Therapeutic Approaches. Medicina.

[15] Caliendo A.M., Gilbert D.N., Ginocchio C.C., Hanson K.E., May L., Quinn T.C., Tenover F.C., Alland D., Blaschke A.J., Bonomo R.A. (2013). Better tests, better care: Improved diagnostics for infectious diseases. Clin. Infect. Dis..

[16] Nash A., Aghlara-Fotovat S., Hernandez A., Scull C., Veiseh O. (2021). Clinical translation of immunomodulatory therapeutics. Adv. Drug Deliv. Rev..

[17] Bashiri A., Halper K.I., Orvieto R. (2018). Recurrent Implantation Failure-update overview on etiology, diagnosis, treatment and future directions. Reprod. Biol. Endocrinol..

[18] Guan D., Sun W., Gao M., Chen Z., Ma X. (2024). Immunologic insights in recurrent spontaneous abortion: Molecular mechanisms and therapeutic interventions. Biomed. Pharmacother..

[19] Cao C., Bai S., Zhang J., Sun X., Meng A., Chen H. (2022). Understanding recurrent pregnancy loss: Recent advances on its etiology, clinical diagnosis, and management. Med. Rev..

[20] Moustakli E., Zikopoulos A., Skentou C., Katopodis P., Domali E., Potiris A., Stavros S., Zachariou A. (2024). Impact of Reductive Stress on Human Infertility: Underlying Mechanisms and Perspectives. Int. J. Mol. Sci..

[21] Ma J., Gao W., Li D. (2022). Recurrent implantation failure: A comprehensive summary from etiology to treatment. Front. Endocrinol..

[22] Zhang S., Lin H., Kong S., Wang S., Wang H., Wang H., Armant D.R. (2013). Physiological and molecular determinants of embryo implantation. Mol. Aspects Med..

[23] Pantos K., Grigoriadis S., Maziotis E., Pistola K., Xystra P., Pantou A., Kokkali G., Pappas A., Lambropoulou M., Sfakianoudis K. (2022). The Role of Interleukins in Recurrent Implantation Failure: A Comprehensive Review of the Literature. Int. J. Mol. Sci..

[24] Sfakianoudis K., Rapani A., Grigoriadis S., Pantou A., Maziotis E., Kokkini G., Tsirligkani C., Bolaris S., Nikolettos K., Chronopoulou M. (2021). The Role of Uterine Natural Killer Cells on Recurrent Miscarriage and Recurrent Implantation Failure: From Pathophysiology to Treatment. Biomedicines.

[25] Kanter J., Gordon S.M., Mani S., Sokalska A., Park J.Y., Senapati S., Huh D.D., Mainigi M. (2023). Hormonal stimulation reduces numbers and impairs function of human uterine natural killer cells during implantation. Hum. Reprod..

[26] Mattar C.N.Z., Chew W.L., Lai P.S. (2024). Embryo and fetal gene editing: Technical challenges and progress toward clinical applications. Mol. Ther. Methods Clin. Dev..

[27] Duan H., Deng W., Kzhyshkowska J., Chen D., Zhang S. (2025). Macrophage at maternal-fetal Interface: Perspective on pregnancy and related disorders. Placenta.

[28] Stavros S., Panagopoulos P., Machairiotis N., Potiris A., Mavrogianni D., Sfakianakis A., Drakaki E., Christodoulaki C., Panagiotopoulos D., Sioutis D. (2024). Association between cytokine polymorphisms and recurrent pregnancy loss: A review of current evidence. Int. J. Gynaecol. Obstet..

[29] Moffett A., Colucci F. (2014). Uterine NK cells: Active regulators at the maternal-fetal interface. J. Clin. Investig..

[30] PrabhuDas M., Piper J.M., Jean-Philippe P., Lachowicz-Scroggins M. (2021). Immune Regulation, Maternal Infection, Vaccination, and Pregnancy Outcome. J. Womens Health.

[31] Jiang X., Wang H. (2020). Macrophage subsets at the maternal-fetal interface. Cell Mol. Immunol..

[32] Wei R., Lai N., Zhao L., Zhang Z., Zhu X., Guo Q., Chu C., Fu X., Li X. (2021). Dendritic cells in pregnancy and pregnancy-associated diseases. Biomed. Pharmacother..

[33] Jameel S., Bhuwalka R., Begum M., Bonu R., Jahan P. (2024). Circulating levels of cytokines (IL-6, IL-10 and TGF- beta) and CD4(+)CD25(+)FOXP3(+)Treg cell population in recurrent pregnancy loss. Reprod. Biol..

[34] Ozbey G., Tanriverdi E.S., Cakir A., Yilmaz E. (2025). Investigation of the Relationship Between IL-17, IL-27, IL-2 Blood Levels in Spontaneous Abortion and Healthy Pregnant Women. Life.

[35] Ding J., Maxwell A., Adzibolosu N., Hu A., You Y., Liao A., Mor G. (2022). Mechanisms of immune regulation by the placenta: Role of type I interferon and interferon-stimulated genes signaling during pregnancy. Immunol. Rev..

[36] Peng X., Chinwe Oluchi-Amaka I., Kwak-Kim J., Yang X. (2025). A comprehensive review of the roles of T-cell immunity in preeclampsia. Front. Immunol..

[37] Sultana S., Nallari P., Ananthapur V. (2020). Recurrent pregnancy loss (RPL): An overview. J. Womens Health Dev..

[38] Catamo E., Zupin L., Segat L., Celsi F., Crovella S. (2015). HLA-G and susceptibility to develop celiac disease. Hum. Immunol..

[39] Andersen L.H.J., Sanz Martinez R., Dai Y., Eriksen J.O., Gerlach M.K., Larsen L.G., Macklon N.S., Juul Hare K., Sandelin A., Nielsen H.S. (2025). Upregulation of immune genes in the proliferative phase endometrium enables classification into women with recurrent pregnancy loss versus controls. Hum. Reprod..

[40] Saito S., Nakashima A., Shima T., Tsuda S. (2021). Pregnancy depends on a delicate balance of immune activation and regulation. Explor. Immunol..

[41] Morelli S.S., Mandal M., Goldsmith L.T., Kashani B.N., Ponzio N.M. (2015). The maternal immune system during pregnancy and its influence on fetal development. Res. Rep. Biol..

[42] Zhang X., Wei H. (2021). Role of Decidual Natural Killer Cells in Human Pregnancy and Related Pregnancy Complications. Front. Immunol..

[43] Canella P., Barini R., Carvalho P.O., Razolli D.S. (2021). Lipid emulsion therapy in women with recurrent pregnancy loss and repeated implantation failure: The role of abnormal natural killer cell activity. J. Cell Mol. Med..

[44] Bustamante J.G., Goyal A., Rout P., Singhal M. (2025). Antiphospholipid Syndrome.

[45] Graham J.J., Longhi M.S., Heneghan M.A. (2021). T helper cell immunity in pregnancy and influence on autoimmune disease progression. J. Autoimmun..

[46] Yockey L.J., Iwasaki A. (2018). Interferons and Proinflammatory Cytokines in Pregnancy and Fetal Development. Immunity.

[47] Sonehara K., Yano Y., Naito T., Goto S., Yoshihara H., Otani T., Ozawa F., Kitaori T., Biobank Japan P., Matsuda K. (2024). Common and rare genetic variants predisposing females to unexplained recurrent pregnancy loss. Nat. Commun..

[48] Zhou Q., Xiong Y., Qu B., Bao A., Zhang Y. (2021). DNA Methylation and Recurrent Pregnancy Loss: A Mysterious Compass?. Front. Immunol..

[49] Cai R., Yang Q., Liao Y., Qin L., Han J., Gao R. (2025). Immune Treatment Strategies in Unexplained Recurrent Pregnancy Loss. Am. J. Reprod. Immunol..

[50] Gunther V., Allahqoli L., Watrowski R., Maass N., Ackermann J., von Otte S., Alkatout I. (2022). Vaginal Microbiome in Reproductive Medicine. Diagnostics.

[51] Blazheva S., Pachkova S., Bodurska T., Ivanov P., Blazhev A., Lukanov T., Konova E. (2024). Unlocking the Uterine Code: Microbiota, Immune Cells, and Therapy for Recurrent Reproductive Failure. Microorganisms.

[52] Balla B., Illes A., Tobias B., Piko H., Beke A., Sipos M., Lakatos P., Kosa J.P. (2024). The Role of the Vaginal and Endometrial Microbiomes in Infertility and Their Impact on Pregnancy Outcomes in Light of Recent Literature. Int. J. Mol. Sci..

[53] Achilli C., Duran-Retamal M., Saab W., Serhal P., Seshadri S. (2018). The role of immunotherapy in in vitro fertilization and recurrent pregnancy loss: A systematic review and meta-analysis. Fertil. Steril..

[54] Bagkou Dimakou D., Tamblyn J., Justin C., Coomarasamy A., Richter A. (2022). Diagnosis and management of idiopathic recurrent pregnancy loss (RPL): Current immune testing and immunomodulatory treatment practice in the United Kingdom. J. Reprod. Immunol..

[55] Turesheva A., Aimagambetova G., Ukybassova T., Marat A., Kanabekova P., Kaldygulova L., Amanzholkyzy A., Ryzhkova S., Nogay A., Khamidullina Z. (2023). Recurrent Pregnancy Loss Etiology, Risk Factors, Diagnosis, and Management. Fresh Look into a Full Box. J. Clin. Med..

[56] Fukui A., Kamoi M., Funamizu A., Fuchinoue K., Chiba H., Yokota M., Fukuhara R., Mizunuma H. (2015). NK cell abnormality and its treatment in women with reproductive failures such as recurrent pregnancy loss, implantation failures, preeclampsia, and pelvic endometriosis. Reprod. Med. Biol..

[57] Brusch A. (2016). The Significance of Anti-Beta-2-Glycoprotein I Antibodies in Antiphospholipid Syndrome. Antibodies.

[58] Liu C., Chu D., Kalantar-Zadeh K., George J., Young H.A., Liu G. (2021). Cytokines: From Clinical Significance to Quantification. Adv. Sci..

[59] Bagkou Dimakou D., Tamblyn J., Lissauer D., Richter A. (2025). Evaluation of peripheral NK tests offered to women with recurrent pregnancy loss and a search for novel candidate biomarkers. J. Reprod. Immunol..

[60] Ali S., Majid S., Niamat Ali M., Taing S., El-Serehy H.A., Al-Misned F.A. (2020). Evaluation of etiology and pregnancy outcome in recurrent miscarriage patients. Saudi J. Biol. Sci..

[61] Ahmad H.I., Jabbar A., Mushtaq N., Javed Z., Hayyat M.U., Bashir J., Naseeb I., Abideen Z.U., Ahmad N., Chen J. (2022). Immune Tolerance vs. Immune Resistance: The Interaction Between Host and Pathogens in Infectious Diseases. Front. Vet. Sci..

[62] Chaplin D.D. (2010). Overview of the immune response. J. Allergy Clin. Immunol..

[63] Kalra A., Mackay O., Thomas-Jones E., Solomon T., Foscarini-Craggs P. (2025). Does the Use of Intravenous Immunoglobulin Improve Clinical Outcomes in Adults With Autoimmune Encephalitis? A Systematic Review. Brain Behav..

[64] Baschieri L., Antonelli A., Nardi S., Alberti B., Lepri A., Canapicchi R., Fallahi P. (1997). Intravenous immunoglobulin versus corticosteroid in treatment of Graves’ ophthalmopathy. Thyroid.

[65] Kumar P., Marron K., Harrity C. (2021). Intralipid therapy and adverse reproductive outcome: Is there any evidence?. Reprod. Fertil..

[66] Skeith L. (2018). Anticoagulating patients with high-risk acquired thrombophilias. Blood.

[67] De Jong P.G., Kaandorp S., Di Nisio M., Goddijn M., Middeldorp S. (2014). Aspirin and/or heparin for women with unexplained recurrent miscarriage with or without inherited thrombophilia. Cochrane Database Syst. Rev..

[68] Li T., Yuan Y., Liu H., Lu Q., Mu R. (2022). Glucocorticoids Improve the Pregnancy Rate and Outcome in Women With Unexplained Positive Autoantibodies: A Systematic Review and Meta-Analysis. Front. Med..

[69] D’Ippolito S., Gavi F., Granieri C., De Waure C., Giuliano S., Cosentino F., Tersigni C., Scambia G., Di Simone N. (2025). Efficacy of Corticosteroids in Patients With Recurrent Pregnancy Loss: A Systematic Review and Meta-Analysis. Am. J. Reprod. Immunol..

[70] Habets D.H.J., Pelzner K., Wieten L., Spaanderman M.E.A., Villamor E., Al-Nasiry S. (2022). Intravenous immunoglobulins improve live birth rate among women with underlying immune conditions and recurrent pregnancy loss: A systematic review and meta-analysis. Allergy Asthma Clin. Immunol..

[71] Han E.J., Lee H.N., Kim M.K., Lyu S.W., Lee W.S. (2021). Efficacy of intralipid administration to improve in vitro fertilization outcomes: A systematic review and meta-analysis. Clin. Exp. Reprod. Med..

[72] Tang Y., Tong X. (2024). Efficacy Evaluation of Aspirin Plus Prednisone or Prednisolone in IVF/RIF Patients: A Systematic Review and Meta-Analysis. Clin. Exp. Obstet. Gynecol..

[73] Ichikawa T., Watanabe T., Kubota Y., Matsuda S., Shigemi D., Kasano S., Yokote R., Yonezawa M., Ouchi N., Negishi Y. (2025). Impact of heparin-aspirin therapy in patients with recurrent pregnancy loss characterized by thrombophilia resistant to low-dose aspirin therapy: A retrospective study. Reprod. Med. Biol..

[74] Yousefi M., Ahmadian-Heris J., Danaii S., Abdolmohammadi-Vahid S., Aghebati-Maleki L., Vladimirov I.K. (2022). Recent Advances in Immunotherapeutic Approaches for Recurrent Reproductive Failure. IVF Technologies and Infertility–Current Practices and New Perspectives.

[75] Sharma P., Goswami S., Raychaudhuri D., Siddiqui B.A., Singh P., Nagarajan A., Liu J., Subudhi S.K., Poon C., Gant K.L. (2023). Immune checkpoint therapy-current perspectives and future directions. Cell.

[76] Romanowska-Prochnicka K., Felis-Giemza A., Olesinska M., Wojdasiewicz P., Paradowska-Gorycka A., Szukiewicz D. (2021). The Role of TNF-alpha and Anti-TNF-alpha Agents during Preconception, Pregnancy, and Breastfeeding. Int. J. Mol. Sci..

[77] Duricova D., Dvorakova E., Hradsky O., Mitrova K., Durilova M., Kozeluhova J., Kohout P., Zarubova K., Bronsky J., Hradska N. (2019). Safety of Anti-TNF-Alpha Therapy During Pregnancy on Long-term Outcome of Exposed Children: A Controlled, Multicenter Observation. Inflamm. Bowel Dis..

[78] McGeachy M.J., Cua D.J., Gaffen S.L. (2019). The IL-17 Family of Cytokines in Health and Disease. Immunity.

[79] Kampan N.C., Xiang S.D., McNally O.M., Stephens A.N., Quinn M.A., Plebanski M. (2018). Immunotherapeutic Interleukin-6 or Interleukin-6 Receptor Blockade in Cancer: Challenges and Opportunities. Curr. Med. Chem..

[80] Vilotic A., Nacka-Aleksic M., Pirkovic A., Bojic-Trbojevic Z., Dekanski D., Jovanovic Krivokuca M. (2022). IL-6 and IL-8: An Overview of Their Roles in Healthy and Pathological Pregnancies. Int. J. Mol. Sci..

[81] Pilat N., Sprent J. (2020). Treg Therapies Revisited: Tolerance Beyond Deletion. Front. Immunol..

[82] Tang C., Hu W. (2023). The role of Th17 and Treg cells in normal pregnancy and unexplained recurrent spontaneous abortion (URSA): New insights into immune mechanisms. Placenta.

[83] Amini L., Kaeda J., Fritsche E., Roemhild A., Kaiser D., Reinke P. (2022). Clinical adoptive regulatory T Cell therapy: State of the art, challenges, and prospective. Front. Cell Dev. Biol..

[84] Rungsiwiwut R., Virutamasen P., Pruksananonda K. (2021). Mesenchymal stem cells for restoring endometrial function: An infertility perspective. Reprod. Med. Biol..

[85] Montgomery L., Larbi A. (2025). Monitoring Immune Responses to Vaccination: A Focus on Single-Cell Analysis and Associated Challenges. Vaccines.

[86] Yang Y., Zhao Y., Liu X., Huang J. (2022). Artificial intelligence for prediction of response to cancer immunotherapy. Semin. Cancer Biol..

[87] Goetz L.H., Schork N.J. (2018). Personalized medicine: Motivation, challenges, and progress. Fertil. Steril..

[88] Jalilvand A., Yari K., Heydarpour F. (2022). Role of Polymorphisms on the Recurrent Pregnancy Loss: A Systematic Review, Meta-analysis and Bioinformatic Analysis. Gene.

[89] Ratre P., Thareja S., Mishra P.K. (2025). Identification of cell-free circulating epigenomic biomarkers for early diagnosis and response to therapies in breast cancer patients. Int. Rev. Cell Mol. Biol..

[90] Felekkis K., Papaneophytou C. (2024). The Circulating Biomarkers League: Combining miRNAs with Cell-Free DNAs and Proteins. Int. J. Mol. Sci..

[91] Yadav R., Singh A.V., Kushwaha S., Chauhan D.S. (2024). Emerging role of exosomes as a liquid biopsy tool for diagnosis, prognosis & monitoring treatment response of communicable & non-communicable diseases. Indian. J. Med. Res..

[92] Adhit K.K., Wanjari A., Menon S., K S. (2023). Liquid Biopsy: An Evolving Paradigm for Non-invasive Disease Diagnosis and Monitoring in Medicine. Cureus.

[93] Benjamin-Davalos S., Koroleva M., Allen C.L., Ernstoff M.S., Shu S. (2021). Co-Isolation of Cytokines and Exosomes: Implications for Immunomodulation Studies. Front. Immunol..

[94] Essola J.M., Zhang M., Yang H., Li F., Xia B., Mavoungou J.F., Hussain A., Huang Y. (2024). Exosome regulation of immune response mechanism: Pros and cons in immunotherapy. Bioact. Mater..

[95] Abdolmohammadi-Vahid S., Danaii S., Hamdi K., Jadidi-Niaragh F., Ahmadi M., Yousefi M. (2016). Novel immunotherapeutic approaches for treatment of infertility. Biomed. Pharmacother..

[96] Wang Y., Tang Z., Teng X. (2024). New advances in the treatment of thin endometrium. Front. Endocrinol..

[97] Mei Y., Lin Y., Chen Y., Zheng J., Ke X., Liang X., Wang F. (2024). Preimplantation genetic testing for aneuploidy optimizes reproductive outcomes in recurrent reproductive failure: A systematic review. Front. Med..

[98] Ebrahimi F., Omidvar-Mehrabadi A., Shahbazi M., Mohammadnia-Afrouzi M. (2024). Innate and adaptive immune dysregulation in women with recurrent implantation failure. J. Reprod. Immunol..

[99] Sathish J.G., Sethu S., Bielsky M.C., de Haan L., French N.S., Govindappa K., Green J., Griffiths C.E., Holgate S., Jones D. (2013). Challenges and approaches for the development of safer immunomodulatory biologics. Nat. Rev. Drug Discov..

[100] Odendaal J., Quenby S., Sammaritano L., Macklon N., Branch D.W., Rosenwaks Z. (2019). Immunologic and rheumatologic causes and treatment of recurrent pregnancy loss: What is the evidence?. Fertil. Steril..

